# Early surgical fixation of proximal femur fractures under active direct oral anticoagulation (DOAC) therapy does not increase the postoperative blood loss. Results from a prospective cohort study with a matched-pair analysis

**DOI:** 10.1007/s00402-025-05870-4

**Published:** 2025-04-12

**Authors:** Valerie Weihs, Michael Humenberger, Géraldine Sturz, Carlo Martin, André Pausch, Andreas Duma, Martin Frossard, Stefan Hajdu

**Affiliations:** 1 Department of Orthopedics and Trauma Surgery – Division of Trauma Surgery, Medical University of Vienna, Vienna, Austria; 2 Department of Anaesthesia, General Intensive Care, and Pain Management – Division of General Anaesthesia and Intensive Care Medicine, Medical University of Vienna, Vienna, Austria; 3 Department of Anaesthesia and Intensive Care Medicine, University Hospital Tulln, Tulln, Austria

**Keywords:** Hip fracture, Proximal femoral fracture, Direct oral anticoagulation, Early surgery

## Abstract

**Introduction:**

This study aims to investigate whether early surgery in patients under active DOAC (direct oral anticoagulation) therapy with trochanteric, proximal femur fractures leads to higher postoperative blood loss.

**Material and methods:**

A prospective cohort study on DOAC patients with trochanteric, proximal femur fractures undergoing early surgery (≤ 24 h) was conducted. Propensity score matching with a retrospective control group of DOAC patients with trochanteric, proximal femur fractures undergoing delayed surgery (> 24 h) was performed for comparison. Key outcome measurements included peri- and postoperative blood loss, transfusion rates, time to surgery and hospital length of stay (LOS).

**Results:**

No differences in the median blood loss in patients undergoing early surgery vs. delayed surgery on postoperative day 1 (1078.35 ml (IQR 745.86) vs. 1120.79 ml (IQR 928.50); *p* = 0.824) or postoperative day 3 (1592.39 ml (IQR 1304.91) vs. 1339.73 ml (IQR 735.57); *p* = 0.165) was seen. No differences in the rate of blood transfusion (72.5% vs. 68.1%; *p* = 0.576) or the number of transfused red blood cells (RBCs) (2 units (IQR 2) vs. 2 units (IQR 3); *p* = 0.567) were detected. A significantly longer median time to surgery, and a significantly longer LOS was seen in the delayed surgery group (*p* < 0.001). No difference in the rates of perioperative complications was detected between both groups.

**Conclusion:**

Early surgery of trochanteric, proximal femur fractures within 24 h under active DOAC therapy does not increase postoperative blood loss or the need for postoperative blood transfusions but leads to a significantly shorter length of stay.

**Level of evidence:**

IIb

## Highlights

• Early surgery of trochanteric, proximal femur fractures does not lead to an increased postoperative blood loss irrespective from active DOAC therapy.

• No increase in the need of postoperative blood transfusions was seen in DOAC patients with early surgery of trochanteric, proximal femur fractures.

• Early surgery of trochanteric, proximal femur fractures leads to significantly shorter length of stays in DOAC patients.

## Introduction

Hip fractures among the elderly population are one of the most common types of fractures and a frequent cause of hospitalization and death [1]. Even though a constant increase in the incidence and the number of hip fracture has already been documented over past decades [2, 3], the number of hip fractures are projected to double within the next 35 years [3, 4]. In addition to this challenge; the increasing incidence of patients with cardiovascular diseases leads to a higher number of patients with active oral anticoagulation therapy worldwide [5]. About 20% of hip fracture patients are under active anticoagulation therapy on admission [6, 7] with substantial comorbidities in more than half of the patients [6]. Hip fracture surgery is recommended within 24 to 48 h after admission [8–11], as a surgical delay has been shown to increase mortality and complications significantly [8, 12–15]. Little is known about the impact of active direct oral anticoagulation (DOAC) therapy on the perioperative bleeding risk among hip fracture patients and the best timing of surgery. Current literature underlines the necessity of expedited surgical protocols in hip fracture patients on DOACs with the opportunity to decrease morbidity and mortality in this group of patients under high risk [16]. Recent international guidelines recommend hip fracture surgery within 24 to 36 h from the last dose of DOAC intake to reduce the percentage of active drugs circulating in the plasma and decrease the risk of perioperative bleeding events [17, 18]. Some reports have shown that fracture fixation within 48 h is only possible in about 40% of DOAC patients [19], in whom longer waiting times to surgery have been associated with an increase in mortality [20]. Despite the lack of prospective trials and conclusive data, no delay of the surgical fixation of hip fractures in DOAC patients is advised [21, 22] as some studies were able to demonstrate no benefit for surgical delay in these patients has been seen [23, 24].

Aiming to partially answer some of these questions, we initiated a prospective study of DOAC patients with trochanteric, proximal femur fractures. As trochanteric, proximal femur fractures have been shown to have the highest perioperative blood loss (mainly driven by the fracture instability and the extracapsular nature of these fractures) [25], our study aimed to investigate if early surgery is feasible irrespective of the DOAC activity. The primary outcome of interest was the perioperative blood loss and rates of needed blood transfusions in DOAC patients with early surgical fracture fixation. The hypothesis of this study was that early surgical fracture fixation does not lead to an increase in the perioperative blood loss or the need of blood transfusions.

## Methods

A prospective, observational cohort study of patients with trochanteric, proximal femur fractures with DOAC therapy undergoing early surgical fracture fixation with a cephalomedullary hip nail within 24 h was performed.

### Study population

Patients with trochanteric, proximal femur fractures under active DOAC therapy have been included.

### Prospective, observational cohort study

Starting in December 2021, a prospective, observational cohort study was initiated. The aim of this study was to investigate whether early (within 24 h after admission) surgical fracture fixation with a cephalomedullary hip nail (early surgery group) leads to an increase in the postoperative blood loss. Patients with trochanteric, proximal femur fractures and active DOAC therapy were considered as eligible for early surgical fixation within 24 h during the study period starting from December 2021. Patients who were not eligible for early surgery (e.g. not eligible for general anesthesia) were not enrolled in the study. From December 2021 to August 2024, 82 consecutive patients were enrolled in the early surgery group (of whom 27 patients have been identified retrospectively).

### Retrospective control group

A retrospective control group of patients with trochanteric fractures under treatment with DOAC medication with delayed surgical fracture fixation with a cephalomedullary hip nail > 24 h after admission from January 2016 to June 2024 was formed (delayed surgery group).

Exclusion criteria included non-active DOAC effect (low, non-therapeutic or non-elevated plasma concentrations) in the early surgery group, surgical delay for other reasons than DOAC therapy in the delayed surgery group, non-surgical management, pathological or non-recent fractures, missing essential data, intraoperative administration of specific reversal agents, multiple fractures, or severe liver dysfunction in both groups.

The primary outcome of interest was the total blood loss on day 1 and day 3 after surgery and the perioperative transfusion rate. Following demographic details were analyzed: sex, age, fracture type (AO classification), height and weight, American Society of Anesthesiologists (ASA) classification, age-adjusted CCI score, time to surgery, peri- and postoperative complications, and the LOS. Blood draws were taken according to our clinical standard on the day of hospital admission, on postoperative day 1 and on postoperative day 3. Laboratory analyses included Haemoglobin (Hb) values as well as plasma concentrations of the oral anticoagulant (anti-Xa assay or dabigatran plasma levels) in the prospective cohort.

The total blood loss was expressed as V_loss_ (ml) and Hb_loss_ (g) from surgery until the first and third postoperative day. The calculations were performed using the “haemoglobin balance method” [26]:

Hb_loss_ = [Blood volume]** X (Hb_a_ – Hb_b_) X 0.001 + Hb_c_

V_loss_ = 1000 X Hb_loss_ /Hb_a_

Hb_a_ (g/l): the hb value prior to surgery

*Hb*_*b*_* (g/l): the Hb value on the third postoperative day (or*,* if not available*,* the 4th post-operative day)*.

*Hb*_*c*_* (g): the total amount of transfused Hb*,* considering that each unit of packed RBC (red blood cell concentrate) contains 55 g of Hb*.

*Blood volume (BV) was calculated using the Lemmens-Bernstein-Brodsky formula [27]

BV = [Blood volume index] X [Weight

*Blood Volume Index (BVi) = 70/sqrt (body mass index (BMI)/22) for males and 65/sqrt (BMI/22) for females.*

The rate of blood transfusions is expressed as the percentage of patients who received at least one unit of RBCs and the total number of transfused RBC units are recorded. The comorbidity assessment was performed using the CCI score [28].

### Statistical analysis

Continuous variables are presented as means and standard deviations or medians and interquartile ranges. Categorical variables are provided with percentages. Descriptive statistics are used for demographic variables and clinical characteristics. The main objective of this study was the evaluation of perioperative blood loss in patients with trochanteric fractures with DOAC medication undergoing early surgical fixation. A chi square test was performed to detect associations of categorical variables. For comparison between categorical variables and continuous variables, the Independent-Samples Median Test and the Mann-Whitney U Test were performed. A two-sided P value of less than 0.05 was considered as statistically significant. Statistical analysis was performed using IBM SPSS Statistics Version 29.0.

**Fig. 1:**
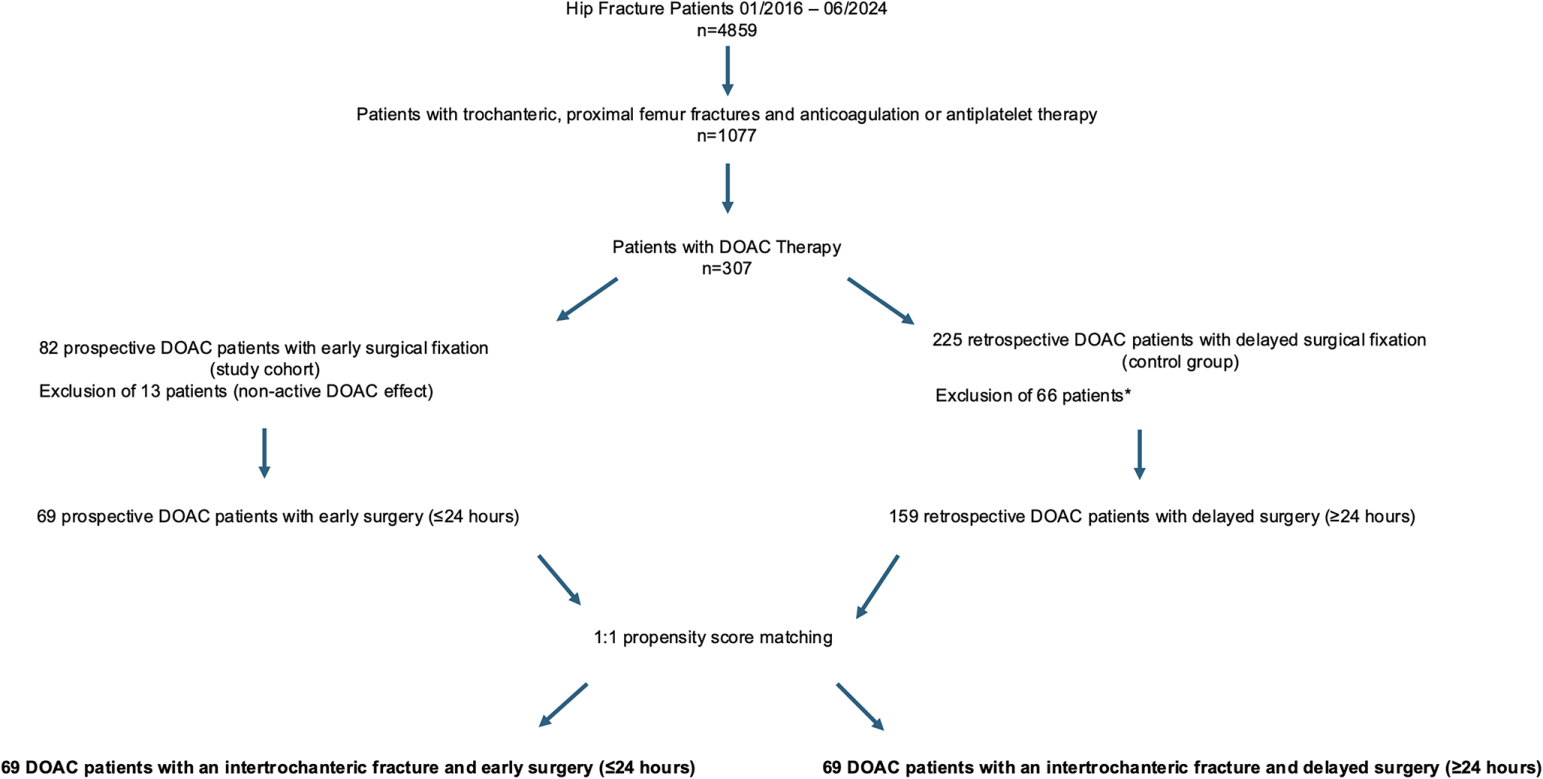
Inclusion of 138 DOAC patients with trochanteric fractures with early surgery (*n* = 69) and delayed surgery(*n* = 69) after 1:1 propensity score matching. *Exclusion of 66 DOAC patients due to: conservative management, surgical delay for other reasons than DOAC therapy, pathological or non-recent fractures; missing data; administration of reversal agents intraoperatively; severe liver dysfunction). DOAC direct oral anticoagulation

The study was designed to achieve 80% power of detecting a statistically significant difference in the primary outcome, while the threshold for type-a error was set to *p* < 0.05. A reduction in total blood loss by 200 ml was considered clinically significant, as it roughly equals a reduction in the plasma level of Hb of 0.5 g/dl for an average adult with 70 kg body weight. Based on existing findings, the estimated blood loss of trochanteric fractures is 1200 ml with a standard deviation in total blood loss of ± 400ml [29]. A sample of at least 64 patients per group was calculated to achieve 80% power. Propensity score matching was performed to address a potential bias by confounding factors in this non-randomized study with a retrospective control group. Covariates that could serve as confounders were defined as age, sex and Charlson-Comorbidity-Index (CCI) score. A 1:1 nearest neighboring matching without replacement was conducted. Appropriate balance was assessed. As a result, out of 228 cases, 138 were successfully matched (Fig. 1). The matching analysis was performed with IBM SPSS statistics Version 29.0.

## Results

Patient demographics and baseline characteristics of the early and delayed surgery group are demonstrated in Table 1.

**Table 1:** *Patient demographics and baseline characteristics. CCI Charlson-Comorbidity index (age-adjusted); ASA American society of anesthesiologists; hb haemoglobin; eGFR estimated glomerular filtration rate. Stable fracture: AO 31A1*, unstable fracture: AO 31A2 and AO 31A3. * Independent-Samples Mann-Whitney U test. ° Chi-squared test.

Variable	Early Surgical Fixation (*n* = 69)	Delayed Surgical Fixation (*n* = 69)	*p*-value
**Sex**,** n (%)**			
Female	44 (63.8%)	44 (63.8%)	1.000°
Median Follow Up, days (IQR)	45 (74)	272.5 (649)	< 0.001*
**Median age**,** yrs (IQR)**	85.33 (9.43)	87.12 (11.24)	0.251*
**Median CCI**,** pts (IQR)**	5.0 (2.0)	6.0 (1.5)	0.059*
**ASA grade**,** n (%)**			
1	/	/	
2	16 (23.9%)	8 (16.0%)	
3	50 (74.6%)	36 (72.0%)	0.046°
4	1 (1.5%)	6 (12.0%)	
**Anesthesia**,** n (%)** General Anesthesia	68 (98.6%)	42 (60.9%)	
Spinal Anesthesia	1 (1.4%)	27 (39.1%)	< 0.001°
**AO Classification**,** n (%)**			
31 A1	31 (44.9%)	24 (34.8%)	
31 A2	31 (44.9%)	34 (49.3%)	0.383°
31 A3	7 (10.4%)	11 (15.9%)	
**Fracture pattern**,** n (%)**			
Stable fractue	31 (44.9%)	24 (34.8%)	
Unstable fracture	38 (55.1%)	45 (65.2%)	0.224
**Median preop Hb g/dl (IQR)**	12.00 (2.15)	11.50 (2.85)	0.183*
**Median Blood Volume ml (IQR)**	4224.50 (966.81)	4092.48 (IQR 1148.60)	0.379*
**Median preop Creatinine g/dl (IQR)**	0.94 (0.39)	1.1 (0.59)	0.018*
**Moderate to severe renal impairment (eGFR < 60mL/min)**,** n (%)**	30 (43.5%)	44 (63.8%)	0.017°
**Median Time to Surgery hours (IQR)**	11.68 (IQR 11.36)	43.22 (IQR 19.75)	< 0.001*

**Fig. 2:**
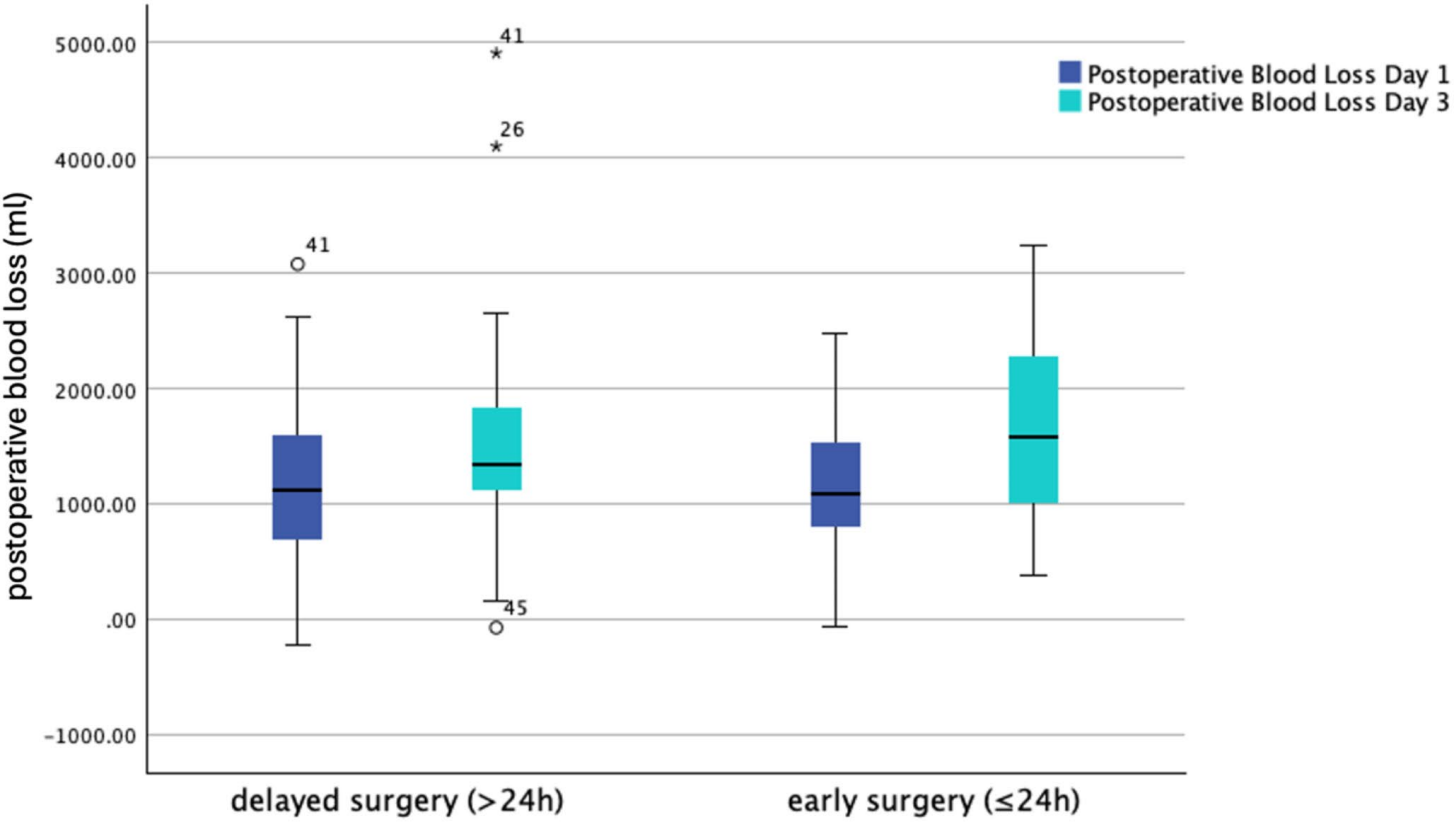
Comparison of median blood loss per group. No significant difference in the median blood loss on day 1 and day 3 after surgery was seen

The overall median blood loss on postoperative day 1 was 1118.60 ml (IQR 843.02) and 1488.86 ml (IQR 865.38) on postoperative day 3. No differences in the median blood loss on postoperative day 1 or postoperative day 3 were seen in both groups, as displayed in Tables 2; Fig. 2. Linear regression analyses revealed no effect of time to surgery (hours) on the postoperative blood loss (ml) on day 1 (95% CI -2.498–4.635; *p* = 0.554) and on day 3 (95% CI -7.265–2.093; *p* = 0.276). A total number of 97 patients (70.3%) required transfusions with at least one unit of RBCs with no difference in the rate of blood transfusion or the number of RBCs between both groups (Table 2)

**Table 2:** Median blood loss on postoperative day 1 and day 3, transfusion rate and median number of transfused RBC per group. IQR interquartile range; RBC red blood Cell. * Independent-Samples Mann-Whitney U test. ° Chi-squared test

Variable	Early Surgical Fixation (*n* = 69)	Delayed Surgical Fixation (*n* = 69)	*p*-value
Median Blood Loss Day 1 ml (IQR)	1078.35 (745.86)	1120.79 (928.50)	0.824*
Median Blood Loss Day 3 ml (IQR)	1592.39 (1304.91)	1339.73 (735.57)	0.165*
Transfusion rate n, %	50 (72.5%)	47 (68.1%)	0.576°
Median transfused RBC units n (IQR)	2 (2)	2 (3)	0.576*

A significantly longer median time to surgery has been recorded in the delayed surgery group (median 43.22 h (IQR 19.75) vs. 11.68 h (IQR 11.36); *p* < 0.001). Patients in the delayed surgery group had significantly longer median LOS (15 days (IQR 5) vs. 11 days (IQR 7); *p* < 0.001).

No differences regarding the rate of in-hospital complications (*p* = 0.110) or the rates of in-hospital deaths (*p* = 0.649) have been seen (Table 3). Revision surgery was needed in 4 patients of the delayed surgery group with a median FU time of 272.5 days (IQR 649) and in 6 patients of the early surgery group with a median FU time of 45 days (IQR 74) without any statistical significance (*p* = 0.511).

**Table 3:** Perioperative complications and in-hospital mortality in both groups showed no statistical significant differences. ° Chi-squared test

Variable	Early Surgical Fixation (*n* = 69)	Delayed Surgical Fixation (*n* = 69)	*p*-value
In-hospital Complication, n (%)	15 (21.7%)	19 (27.5)	0.110*°*
Urinary tract infection, n (%)	1 (1.4%)	7 (10.1%)	0.163°
In-Hospital Death, n (%)	2 (2.9)	3 (4.3%)	
Other, n (%)	6 (8.7%)	5 (7.2%)	

## Discussion

In the next decades, a constant increase in the number of hip fractures is expected [3, 4] with a concordant increase in patients under active anticoagulation therapy. There is still an ongoing debate about the optimal timing of hip fracture surgery in patients under active anticoagulation therapy [16, 19–24]. The total blood loss in hip fracture surgery depends on the surgical treatment, with the greatest perioperative blood loss occurring with the use of intramedullary hip nails and dynamic hip screws [25]. To reduce intra- and postoperative blood loss and allow potential medical optimization, recent international guidelines recommend hip fracture surgery in DOAC patients within 36 h, balancing the risk of potential bleeding complications against the overall risks of delaying surgery [17].

Existing literature suggests, that early hip fracture surgery seemed to be safe [19, 21–24] with potential beneficial effects on the mortality of these patients [30, 31]. So far, most studies were quite heterogenous, mainly from a retrospective nature and demonstrated varying results with regards to the perioperative blood loss. Schuetze et al. demonstrated that early surgery of proximal femur fractures in DOAC patients leads to an increased risk for intraoperative blood transfusion [32], whereas Krespi et al. found no influence on postoperative blood loss [31]. To overcome these contradictory results, our study aimed to present a homogenous study group with trochanteric femoral fractures in patients with proven drug activity prior to surgical intervention.

Firstly, our data suggests that early surgery of trochanteric femur fractures with a median time to surgery of about 12 h does not increase the perioperative blood loss in DOAC patients. Even though, the early surgical fixation group surpassed the 200 ml mark of blood loss on day 3, predefined as a value of clinical significance, neither any statistically significant difference in the overall postoperative blood loss nor higher rates of RBC transfusion have been detected. Furthermore, no effect of early surgery on the postoperative blood loss on day 1 or day 3 could be seen suggesting that early surgery within 24 h is feasible with regard to the postoperative blood loss.

Secondly, we found significantly shorter LOS in the early surgery group. The waiting time to surgery in the delayed group of our study cohort (median time to surgery 43 h) was consistent with current evidence and guidelines for the treatment of patients with hip fractures [8–11]. We noted a trend towards a higher comorbidity burden in the delayed group that might have an additional effect on the LOS besides the longer waiting time to surgery. Additionally, patients in the delayed cohort showed higher rates of renal dysfunction. This might have prolonged the waiting time to surgery as international guidelines recommend longer waiting times to surgery in DOAC patients with impaired renal function [17, 18]. Comorbidities and pre-injury anticoagulant use seem to be responsible for the majority of delayed hip fracture surgeries [33]. As already mentioned, a surgical delay of hip fracture surgery has been shown to be closely linked to increased mortality and complications rates [8, 12–15]. Consistent with recent literature early surgery resulted in our study cohort in significantly shorter LOS and did not increase the in-hospital complication rates. Globally the burden of hip fractures is constantly increasing [1–4] and a shortening of the LOS results in lowering the economic burden without increasing mortality or complication rates [34]. As reported recently, approximately 1/3 of hip fracture patients will undergo delayed surgery due to comorbidities and there is small subgroup of patients that might benefit from medical optimization prior to surgery [33, 35]. Given the high comorbidity burden in both of our study groups and the similar in-hospital complication rates, most DOAC patients may benefit from early trochanteric hip fracture surgery.

### Limitations of this study

The study was designed to detect a potential statistical difference in the postoperative bleeding rates after early surgery which led to a relatively small sample size in concordance with the a-priori power analysis. Therefore, the power of this specific study may not be sufficient to perform any sub-analysis of the potential benefit of early surgery on the mortality of this patients. The long-term follow-up and long-term mortality rates were not included in the data analysis since a follow-up of at least 90 days up to 1 year would be required to assess the benefit of early surgery on mortality. These limitations impact the generalizability of our findings as the impact of early surgery on the long-term outcome or mortality is still unknown. Other limitations of this study include the potential dehydration of our patients on admission, which might affect the preoperative Hb levels. As this limitation applies to both groups, it should not affect the statistical analysis. Additionally, the inclusion of retrospective DOAC patients during the Covid-19 pandemic might serve as potential bias, although care has been taken to exclude all retrospective DOAC patients with a delay of surgery due to other reasons than the anticoagulation therapy. Future research is needed to find the optimal timing of surgery for intracapsular, proximal femur fractures in DOAC patients.

In conclusion, this prospective, observational cohort study showed that early surgical fixation within 24 h of trochanteric, proximal femur fractures under active DOAC therapy does not increase perioperative blood loss or the need for postoperative blood transfusions.

According to our results, early surgical fixation is recommended due to a significantly shorter length of hospital stay without increasing perioperative bleeding rates or the rates of perioperative complications.

## Data Availability

No datasets were generated or analysed during the current study.

## Declarations

### Data sharing

The datasets generated and analyzed in the current manuscript are not publicly available due to data protection regulations. The data used and analyzed in this study are available from the corresponding author solely upon reasonable request.
